# Primary CNS vasculitis (PCNSV): a cohort study

**DOI:** 10.1038/s41598-022-17869-7

**Published:** 2022-08-05

**Authors:** Ayush Agarwal, Jyoti Sharma, M. V. Padma Srivastava, M. C. Sharma, Rohit Bhatia, Deepa Dash, Vinay Goyal, Achal K. Srivastava, Manjari Tripathi, Vaishali Suri, Mamta B. Singh, Sushant Agarwal, Chitra Sarkar, Leve Joseph, Manmohan Singh, Ashish Suri, Rajesh K. Singh, Deepti Vibha, Awadh K. Pandit, Roopa Rajan, Anu Gupta, A. Elavarasi, Divya M. Radhakrishnan, Animesh Das, Shailesh Gaikwad, Vivek Tandon, Ramesh Doddamani, Ashish Upadhyay, Ajay Garg, Venugopalan Y. Vishnu

**Affiliations:** 1 Department of Neurology, All India Institute of Medical Sciences, New Delhi, India; 2 Department of Neuropathology, All India Institute of Medical Sciences, New Delhi, India; 3 Department of Neuroradiology, All India Institute of Medical Sciences, New Delhi, India; 4 Department of Neurosurgery, All India Institute of Medical Sciences, New Delhi, India; 5 Department of Biostatistics, All India Institute of Medical Sciences, New Delhi, India

**Keywords:** Neuroscience, Neurology

## Abstract

Primary CNS Vasculitis (PCNSV) is a rare inflammatory disorder affecting the blood vessels of the central nervous system. Patients present with a combination of headaches, seizures, and focal neurological deficits. There is usually a diagnostic delay. Treatment is based on observational studies and expert opinion. Our objective was to identify clinical, laboratory, neuroimaging, pathologic or management-related associations with 2 year outcome in patients with primary CNS vasculitis. We conducted a cohort study at a single tertiary care referral centre of prospectively (2018-2019) and retrospectively (2010-2018) identified individuals with primary CNS vasculitis (diagnosis was proven by either brain biopsy or cerebral digital subtraction angiography). Clinical, imaging and histopathologic findings, treatment, and functional outcomes were recorded. Univariate and stepwise multiple logistic regression were applied. P-value<0.05 was considered statistically significant. The main outcome measures were documentation of clinical improvement or worsening (defined by mRS scores) and identification of independent predictors of good functional outcome (mRS 0-2) at 2 years. We enrolled eighty-two biopsy and/or angiographically proven PCNSV cases. The median age at presentation was 34 years with a male predilection and a median diagnostic delay of 23 months. Most patients presented with seizures (70.7%). All patients had haemorrhages on MRI. Histologically lymphocytic subtype was the commonest. Corticosteroids with cyclophosphamide was the commonest medication used. The median mRS at follow-up of 2 years was 2 (0-3), and 65.2% of patients achieved a good functional outcome. Myelitis and longer duration of illness before diagnosis were associated with poorer outcomes. The presence of hemorrhages on SWI sequence of MRI might be a sensitive imaging marker. Treatment with steroids and another immunosuppressant probably reduced relapse rates in our cohort. We have described the third largest PCNSV cohort and multi-centre randomised controlled trials are required to study the relative efficacy of various immunosuppressants.

**Study registration:** CTRI/2018/03/012721.

## Introduction

Primary CNS Vasculitis (PCNSV) is a rare, heterogeneous and polymorphic inflammatory disorder affecting the blood vessels of the central nervous system, often resulting in significant morbidity and mortality^1,2^. It has a male preponderance with a median onset of 50 years^1,3,4^. Patients usually present with a combination of headaches, seizures and focal neurological deficits, and there is a long time lag between symptom onset and eventual diagnosis^3–5^. The diagnosis is made by the Calabrese and Mallek criteria^6^, where an unexplained acquired neurological deficit with either an angiogram or CNS biopsy shows features of vasculitis, with other causes ruled out and requires a high index of suspicion.

Even though a plausible diagnosis of PCNSV may be made on MRI correlating with clinical findings and cerebral DSA, meningo-cortical/spinal cord biopsy remains the reference standard to certify the diagnosis. The treatment of PCNSV is based on retrospective studies and expert opinions since there have been no RCTs. Till date, only a few adult cohorts have been reported, the largest ones by Salvarani et al. from Mayo clinic^4^ and de Boysson et al. from the French cohort^5^. We describe the third largest cohort of adult PCNSV, including clinical demographics, management and predictors of a good functional outcome at 2 years.

## Methods

### Patient recruitment

From January 1, 2010, through July 31, 2019, patients at AIIMS, New Delhi who were diagnosed with PCNSV were included in the study. We initiated the PCNSV registry after approval by the All India Institute of Medical Sciences Institutional Ethics Committee in February 2018, and the protocol is registered in the Clinical Trial Registry of India (CTRI/2018/03/012721). Informed consent was obtained from all subjects or their legal guardians. All cases before the registry were retrieved retrospectively from the files and electronic hospital records. All clinical data were verified by two neurologists. The age of onset was considered to be the age at which the first clinical features were apparent. We followed the EQUATOR reporting guidelines and the study was conducted in accordance with the declaration of Helsinki and principles of good clinical practice.

### Inclusion criteria

All adult patients (age ≥ 18 years) with a diagnosis of PCNSV, satisfying the diagnostic criteria below:

1. Recent history or presence of an acquired unexplained neurological deficit,
2. Evidence of vasculitis in a CNS biopsy specimen, or
3. Cerebral DSA with changes characteristic of vasculitis.

### Exclusion criteria

1. Evidence of systemic vasculitis or other PCNSV mimickers.
2. Hypercoagulable state.
3. Findings explained by other causes.

### Blood investigations

Serum vasculitis profile (ANA, lupus anticoagulant, anti-cardiolipin antibody, anti-dsDNA and ANCA) was done in all patients to exclude a systemic vasculitis. Common chronic infectious inflammatory aetiologies were ruled out by testing for HIV, hepatitis B and C, and syphilis (VDRL). Other tests like HLA-B51, RA factor, anti-SSA and SSB, cryoglobulins and 2D-echocardiography were done based on patient’s clinical profile. Serum NMO and MOG antibody testing was done in all patients with spinal cord manifestations.

### Imaging studies

MRI and DSA were reviewed and analysed by two neuroradiologists. MRIs were reviewed for abnormalities in the form of grey and white matter changes, haemorrhages, and enhancing lesions in the different CNS regions^7–9^.

DSAs were evaluated for arterial (steno-occlusive changes, irregularity, beaded/pearlescent appearance, aneurysms) or venous phase abnormalities (dilatation and tortuosity of small veins, puddling and staining of contrast, parenchymal venous phase abnormalities).

### Histopathology

Biopsies were either targeted (sampled from regions of the brain that demonstrated abnormalities on imaging) or blind (non-dominant frontal or temporal pole). They were reviewed and reported by the neuropathologist, and a PCNSV diagnosis required the presence of transmural inflammation of small- or medium-sized meningo-cortical blood vessels. It was further classified into granulomatous, lymphocytic and necrotizing subtypes. Patients with both biopsy and angiography positive PCNSV were considered as biopsy positive cases.

### Cerebrospinal fluid (CSF)

CSF examination was considered abnormal if cells > 5/µL or protein > 45 mg/dL.

### Management strategies and functional outcome

The treatment received was documented and good clinical outcome was defined as an mRS score of 0–2. Relapse was defined as a new clinical deficit, an increase in the pre-existing symptoms, or evidence of a new lesion on follow up MRI. However, seizure or headache recurrence without new MRI lesions did not qualify as relapse.

### Statistical analysis

Categorical variables were reported as proportions, whereas continuous variables as medians with interquartile range. Wherever applicable, differences in categorical variables were assessed by chi-square or Fisher’s exact test, whereas continuous variables were assessed by Mann Whitney test. Univariate and step-wise multiple logistic regression was applied to find independent predictors of good functional outcome (mRS 0–2), and adjusted Odds ratio were calculated. P-value < 0.05 was considered statistically significant. All statistical analyses were done using SPSS version 27.0.

## Results

**Figure 1:**
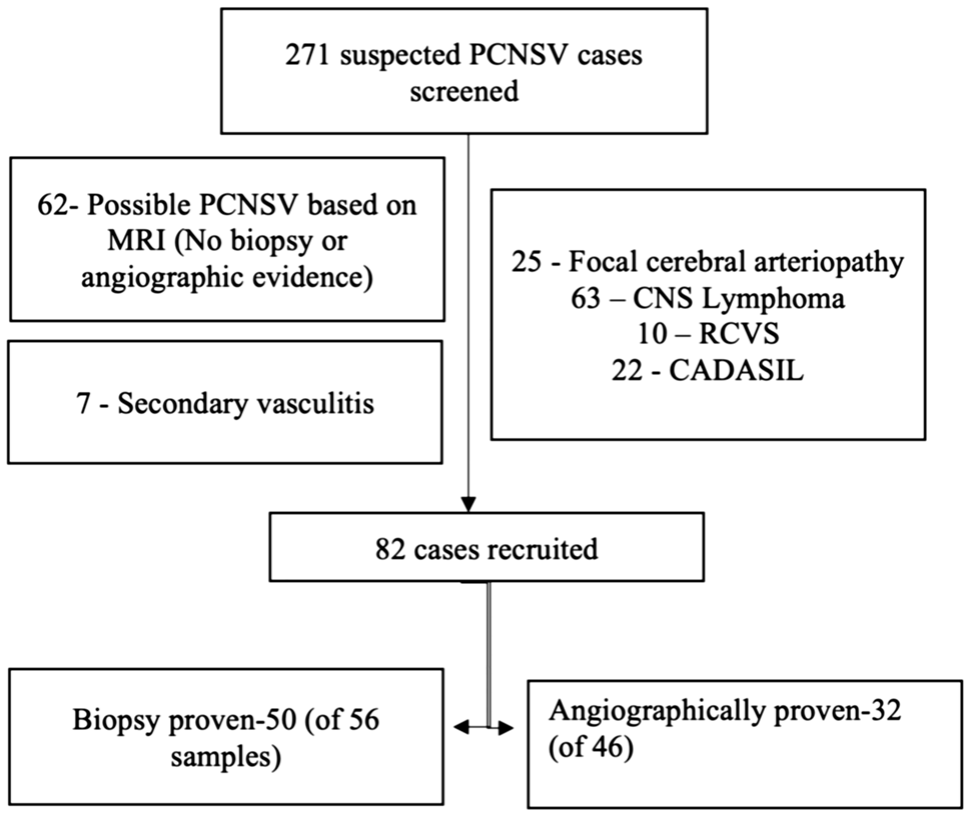
Patient screening and selection for our study.

A total of 271 patients with suspected PCNSV were screened, and 82 cases were identified as per inclusion and exclusion criteria. Fifty had a positive biopsy, forty-five had a positive DSA, and thirteen had dual positivity (Fig. 1).

The clinical characteristics are mentioned in Table 1. The median age at presentation was 34 years with a male predilection (4.8:1), and there was a median diagnostic delay of 23 months from the first symptom to diagnosis. The most common presenting symptom was seizures (70.7%), followed by headache (59.8%). Most of our patients suffered from progressive deficits. Concomitant hypertension was present in 13 patients and diabetes in 4 patients. ESR was elevated in 27 patients (32.9%).

**Table 1:** Clinical characteristics.

	PCNSV cohort (n = 82)
Sex (male:female)	68:14 (4.8:1)
Median age at presentation (IQR)	34 (27.8–42) years
Median age at onset (IQR)	28.6 (22.9–38.2) years
Median age at diagnosis (IQR)	32 (25–39.2) years
Diagnostic delay	23 (6.8–48) months
Symptoms [number(%)]	Seizures—58 (70.7%) Headache—49 (59.8%) Hemiparesis—45 (54.9%) Cognitive impairment—24 (29.3%) Visual impairment—18 (21.9%) Ataxia—17 (20.7%) Paraparesis—11 (13.4%) Fever—0
Clinical course_1	Static—9 (10.9%) Progressive—73 (89.1%)
Clinical course_2	Monophasic—15 (18.3%) Multiphasic—67 (81.7%)
Relapse	10 (12.2%)
Lost to follow up	36 (43.9%)
Favourable mRS 0–2	Yes—30/46 (65.2%)
**mRS distribution**	
mRS0	19
mRS1	3
mRS2	8
mRS3	7
mRS4	3
mRS5	2
mRS6	4
Mortality	4/46 (8.7%)
Initial treatment	Steroids only—5 (6.2%) Steroids + cyclophosphamide—40 (48.8%) Steroids + azathioprine—24 (29.3%) Steroids + methotrexate—3 (3.6%) Steroids + mycophenolate—3 (3.6%) Steroids + rituximab—7 (8.5%)
Treatment at last follow up	Steroids +/− ASD—3 (6.5%) Steroids + immunosuppresants +/− ASD—7 (15.2%) Immunosuppressants +/− ASD—13 (28.3%) Antiseizure drugs (ASD) only—13 (28.3%) No treatment—6 (13%) Dead—4 (8.7%)

*CSF examination* was done in 48/82 patients and was abnormal in 42 (87.5%); pleocytosis in 21 and increased protein in 42 (median value-82.5 mg/dl; range: 29-444 mg/dl). Gram’s stain, TB-PCR, VDRL, Cryptococcal antigen, and cultures were negative in all patients (Supplementary Table 1).

### Imaging

The brain as the first CNS region involved was seen in 85.4% cases, spinal cord in 6.1%, and both in 8.5% cases (Supplementary Table 1). Microhemorrhages and/or macrohemorrhages were seen in all the patients. Spinal cord involvement was commonly multifocal and dorsal cord was most frequently involved.

Cerebral DSA was done in 46/82 patients, and was abnormal in 45 patients (97.8%). Most patients (41/45) with abnormal DSA had venous phase abnormality. Four patients had arterial abnormalities (2 had multiple arterial irregularities with vessel occlusions, and 2 had multiple arterial irregularities only).

### Histopathology

A meningo-cortical biopsy was done in 56 out of the 82 patients (targeted:49, blind:7) and showed features of PCNSV in fifty (89.3%) patients. Though targeted biopsy had a higher positivity rate than blind biopsy, the difference was not statistically significant.

**Figure 2:**
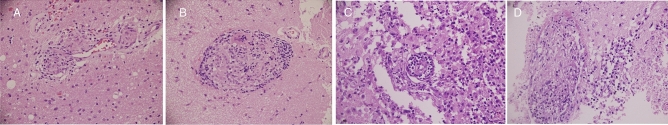
Photomicrographs showing non-necrotizing granulomas involving the vessel wall on Hematoxylin and Eosin stain at 200 × (**A**) and 400 × (**B**) respectively. Photomicrographs showing lymphocytic vasculitis (**C**) and thrombus formation in the vessels (**D**).

Lymphocytic vasculitis was the most common PCNSV subtype (54%), followed by granulomatous vasculitis (44%) and necrotizing vasculitis (2%) (Fig. 2).

### Management

The most common treatment modality used was steroids with cyclophosphamide [intravenous 750 mg/m^2^, once a month for 6 doses] (n = 40) followed by steroids with azathioprine [2–3 mg/kg/day] (n = 24). Other immunosuppressants used were mycophenolate mofetil (2–3 gm/day) (n = 3), methotrexate (15–25 mg/week) (n = 3), and rituximab (375 mg/m^2^ intravenous infusion once a week for four weeks followed by a repeat infusion at six months, if required) (n = 7). Two patients required additional treatment with intravenous immunoglobulin. The 2 year mRS scores were available in 46 patients with the median mRS score being 2 (IQR 0–3). Thirty patients (65.2%) achieved a good functional outcome (mRS 0–2); 10 cases had a relapse, while four of our patients died (Table 1).

The presence of delay in diagnosis of PCNSV (p—0.007), paraparesis (p—0.000) and spinal cord involvement by disease (p—0.001) were adversely associated with functional outcomes on univariate analysis (Table 2). Multivariate analysis revealed spinal cord involvement (OR 0.04, CI 0.004–0.392) and delay in diagnosis (OR 0.31, CI 1.15–10.92) to be associated with worse outcomes (Table 2).

**Table 2:** Predictors of favourable functional outcome (mRS 0–2).

Univariate analysis	Patients with mRS 0–2 (n = 30)	Patients with mRS 3–6 (n = 16)	p-value
Sex (M:F)	26:4	13:3	0.626
Median age (IQR)	35.5 (29–41) years	33 (29–44) years	0.431
Median symptom onset age (IQR)	34 (25–39) years	28.4 (26.4–38.7) years	0.336
Age at diagnosis	34.8 (28–40) years	32.1 (29.1–41.7) years	0.455
Delay in diagnosis	11 (6–24) months	39 (21–51.5) months	0.007
Headache	21	8	0.181
Cognitive impairment	9	4	0.720
Hemiparesis	15	9	0.686
Progressive course	28	16	0.291
Multiphasic course	25	13	0.859
Paraparesis	0	7	0.000
Ataxia	5	5	0.253
Seizure	21	8	0.181
Visual impairment	5	6	0.115
Abnormal CSF protein	19	13	0.805
CSF leucocytosis	12	5	0.214
Spinal cord involvement by disease	2	9	0.001
Hemorrhages	30	16	0.702
Granulomatous PCNSV	9	2	0.075
Lymphocytic PCNSV	9	6	0.081

Multivariate analysis	Odds ratio	p-value	Confidence interval
Spinal cord involvement	0.04	0.006	0.004–0.392
Delay in diagnosis	0.31	0.031	1.15–10.92

## Discussion

The median age of onset was 28.6 years, significantly younger than the other major cohorts with male predominance (82.9%)^4,5,10^. Our patients were younger as compared to other Western cohorts^4,5^ and Europe but was similar to the series from the southern part of India (Sundaram et al.)^10^. Vasculitis may affect an individual of any age. The reason for this, although not clearly understood, probably reflects an interaction between an unknown environmental trigger or a genetic predisposition. The sex ratio was skewed towards males probably because of a region-specific male bias towards seeking tertiary care.

Other major comparatives between our study and these 3 cohorts are listed in Table 3. CSF was abnormal in approximately 90% of our patients which advocates for CSF analysis in suspected PCNSV cases, especially to rule out infectious causes since remaining findings are non-specific and may non-contributory.

**Table 3:** Comparison between the largest cohorts of PCNSV.

	Mayo cohort^4^	French cohort^5^	Sundaram et al.^10^	AIIMS cohort
Total cases	163	112	45	82
Median age (years)	48	47	36	28.6
Sex (M:F)	78:89	60:52	31:14	68:14
Most common symptom	Headache (59.5%)	Motor deficit	Hemiparesis	Seizures (70.7%)
Abnormal DSA	113/163 (69.3)	74/95 (78)	30/42 (71)	45/46 (97.8)
Abnormal biopsy (%)	58/81 (72)	33/53 (62)	19/29 (66)	50/56 (89.3)
Abnormal CSF (%)	117/126 (93)	73/104 (70)	25/45 (60)	42/48 (87.5)
Median mRS		2	2	2
Good functional outcome	mRS 0–3	mRS 0–2: 63/112 56%	mRS 0–2: 33/45 77%	mRS 0–2: 30/46 65.2%
Mortality (%)	25/163 (15)	9/112 (8)	7/45 (16)	4/46 (8.5%)

MRI was abnormal in all cases. In a systematic review of published cases of PCNSV, cerebral MRI was anomalous in 93% of patients^11^ and CSF was abnormal in 74%^12^. In our study, SWI showed hemorrhages in 100% PCNSV cases, which is significantly high compared to the existing literature. This could be due to selection bias as these patients might have been selectively taken up for DSA or brain biopsies. The most frequent presentation in PCNSV is brain hemorrhage with an incidence of 10.8–63.6% on MRI^8,13,14^. Hemorrhages have been reported to be commoner in women and associated with necrotizing vasculitis subtype^14^.Our results might indicate that hemorrhages are underestimated in other studies and the increased incidence in our study might be due to the use of SWI in all cases. Post-treatment with steroids, the white matter FLAIR hyperintensities may disappear, but the SWI hemorrhages remain^15^.

We found evidence of spinal cord involvement in 38.3% which was significantly higher than 5% documented by Salvarani et al. (2008d). We concurred that the thoracic cord was most frequently involved. This was probably due to the institute policy of imaging the spinal cord in all patients with suspected PCNSV.

DSA was done in suspected cases based on MRI findings. It showed a high sensitivity to detect vasculitis and was positive in 98% cases. Lack of a high prevalence of arterial abnormalities in our cohort could be due to predominant small vessel vasculitis in our cohort (all small-vessel vasculitis are not detectable by DSA because its resolution cannot discern vessel diameters < 0.2 mm), secondary to case selection and lack of DSA in all cases. DSA has a sensitivity of 50–90% in the diagnosis of PCNSV^16–19^. CTA/MRA, though not well studied with histopathological confirmation, appear even less sensitive^20–22^. Other inflammatory, metabolic, malignant or vascular pathologies can simulate PCNSV like ICAD, RCVS, radiation vasculopathy and should always be ruled out.

CNS biopsy was positive in approximately 90% of our cases which is the highest amongst cohorts reported. Most of these were targeted biopsies. Lymphocytic subtype was the most common (54%). The lymphocytic subtype was the most common in the series by Sundaram et al.^10^ and Oon et al.^23^, while granulomatous subtype was the most common in the Mayo cohort^4^. None of our patients had findings of amyloid angiopathy on histopathology.

Most patients were treated with steroids in combination with cyclophosphamide or azathioprine. A small proportion of patients were also treated with a combination of steroids with rituximab, methotrexate, mycophenolate or steroids alone. This highlights the lacunae of PCNSV treatment which till date is guided by expert opinion and retrospective data since RCTs are lacking. Ten of our patients had a relapse on treatment. However, no variable was significantly associated with relapse occurrence. Relapses may affect between 30 and 50% cases^4,24,25^ and the lesser relapses in our cohort confirms that an induction strategy with steroids and a potent immunosuppressant is beneficial in supressing the initial inflammatory process and preventing subsequent flare-ups. Cyclophosphamide was the most commonly used immunosuppressant (48.8%) as has been recommended by the European League Against Rheumatism for small and medium vessel vasculitis^26^. Rituximab was also used in a substantial number of our patients (8.5%) as it is a good alternative to cyclophosphamide in patients with contraindications, toxicity or reservations for use^27,28^. No treatment modality was associated with a significantly better outcome at follow-up.

Most of our patients were off steroids on follow-up (69.6%), requiring either no treatment (13%), antiseizure drugs alone (28.3%), or an immunosuppressant (28.3%). There was no significant difference between the biopsy-proven and angiographically proven cases (Supplementary Table 2).

A good functional outcome was taken as an mRS between 0 and 2. A 2 year follow-up data was available for 46 of our patients. The median mRS was 2, and good functional outcome was achieved by 65.2% of our cases. We lost four patients (8.7%). These outcome measures were similar to other previously published PCNSV cohorts^4,5,10^ (Table 3). Spinal cord involvement and delay in diagnosis were associated with poorer outcomes.

The strength of present study is the large number cases with long follow-up which helped to confirm the diagnosis and rule out mimics. Our cohort of PCNSV is the third-largest in the world and adds to literature of this rare disease entity. We included only those cases which were diagnosed angiographically or by CNS biopsy, with all potential mimickers excluded. We describe the largest number of PCNSV cases affecting the spinal cord. Compared with individual case reports or smaller previous series, our study cohort is largest in which SWI has been done.

Our study has various limitations. Its partial retrospective nature binds it to the shortcomings of data collection, possible selection bias and potential confounding factors. However, data was collected prospectively after initiation of the PCNSV registry in February 2018. The imaging studies were performed on different scanners of variable strength and heterogeneous protocols. All of our patients didn’t undergo DSA, which is taken as the reference-standard for the prebiopsy diagnosis of PCNSV^29^. CNS biopsy is considered the gold standard for PCNSV diagnosis but was not done in all cases. A significant number of patients were lost to follow up after first discharge.

## Conclusion

We describe a cohort PCNSV patients which showed the utility of DSA and brain biopsy in achieving final diagnosis. The presence of micro and macrohemorrhages in SWI sequence on MRI Brain might be a good imaging marker for PCNSV. Treatment with steroids and another immunosuppressant probably reduced relapse rates in our cohort. Multi-centre RCTs are required to find the relative efficacy of various immunosuppressants.

## Supplementary Information

Supplementary Tables.

## Data Availability

The datasets used and/or analysed during the current study available from the corresponding author on reasonable request.

## Abbreviations

AIIMS: All India Institute of Medical Sciences
ANA: Anti-nuclear antibody
ANCA: Antineutrophilic cytoplasmic antibodies
Anti-ds DNA: Anti-double stranded deoxyribonucleic acid
Anti-SSA and SSB: Anti-Sjogren’s syndrome-related antigen A and B
CNS: Central nervous system
CSF: Cerebrospinal fluid
CTA: Computed tomographic angiography
DSA: Digital subtraction angiography
ESR: Erythrocyte sedimentation rate
FLAIR: Fluid attenuated inversion repeat
HIV: Human immunodeficiency virus
ICAD: Intracranial atherosclerotic disease
ICH: Intracerebral hemorrhage
IQR: Interquartile range
MOG: Myelin oligodendrocyte glycoprotein
MRA: Magnetic resonance angiography
MRI: Magnetic resonance imaging
mRS: Modified Rankin scale
NMO: Neuromyelitis optica
PCNSV: Primary central nervous system vasculitis
RA: Rheumatoid arthritis
RCT: Randomised controlled trial
RCVS: Reversible cerebral vasoconstriction syndrome
SWI: Susceptibility weighted imaging
TB-PCR: Tuberculosis-polymerase chain reaction
VDRL: Venereal disease research laboratory
