# Supplementary material for: Primary CNS vasculitis (PCNSV): a cohort study
Source: Sci Rep. 2022 Aug 5;12:13494. doi: 10.1038/s41598-022-17869-7 (PMC9355950; doi:10.1038/s41598-022-17869-7)
Supplement: Supplementary file 1 — Supplementary Tables. [file 41598_2022_17869_MOESM1_ESM.docx]

**Supplementary table 1: Investigations**

| **CSF examination**   - Done- 48 (58.5%) - Not done- 34 (41.5%) | **Abnormal- 42/48 (87.5%)**   - Abnormal cells- 21 (43.8%) - Abnormal protein- 42 (87.5%) | |
| --- | --- | --- |
| **DSA**   - Done- 46 (56.1%) - Not done- 36 (43.9%) | **Abnormal- 45/46 (97.8%)**   - Unilateral involvement- 2 (4.4%) - Bilateral involvement- 43 (95.6%) - Arterial irregularities- 4 (8.9%)  1. Beaded appearance- 4 2. Vessel occlusion- 2  - Venous phase abnormality- 41 (91.1%) | |
| **Biopsy**   - Done- 56 (68.3%)  1. Blind frontal- 6 2. Blind temporal-1 3. Targeted- 49  - Not done (31.7%) | **Abnormal- 50/56 (89.3%)**   - Granulomatous- 22 (44%) - Lymphocytic- 27 (54%) - Necrotizing- 1 (2%) | |
| **MRI**   - Done- 82 (100%) | Median number of MRI studies (IQR)  First CNS region involved  Brain regions involved  Hemorrhages | 3 (2-5)  Brain- 70 (85.4%)  Spinal cord- 5 (6.1%)  Both- 7 (8.5%)  Supratentorial only- 14 (17.1%)  Infratentorial only- 1 (1.2%)  Both- 67 (81.7%)  Microhemorrhages only- 35 (42.7%)  Macrohemorrhages only- 0  Both- 47 (57.3%) |

**Supplementary table 2: Comparison between biopsy proven and angiography proven PCNSV patients**

|  | **Biopsy proven (n=50)** | **Angio proven (n=32)** | **p-value** |
| --- | --- | --- | --- |
| Sex (M:F) | 40:10 | 29:3 | 0.199 |
| Median age at presentation (IQR) | 34 (28-44) years | 34 (28-39.5) years | 0.749 |
| Median age at symptom onset | 29.9 (22.6-38.7) years | 28.3 (25-34.4) years) | 0.728 |
| Median age at diagnosis | 32.5 (25-40) years | 32 (26.5-38.2) years | 0.594 |
| Diagnostic interval | 17.5 (6-36) months | 30 (11.5-54) months | 0.059 |
| Headache | 31 | 18 | 0.604 |
| Hemiparesis | 24 | 21 | 0.118 |
| Cognitive impairment | 17 | 7 | 0.239 |
| Progressive course | 45 | 28 | 0.724 |
| Multiphasic course | 43 | 24 | 0.209 |
| Paraparesis | 6 | 5 | 0.638 |
| Ataxia | 9 | 8 | 0.446 |
| Seizure | 36 | 22 | 0.752 |
| Visual symptoms | 11 | 6 | 0.723 |
| CSF done | 26 | 22 | 0.124 |
| Abnormal CSF protein | 23 | 19 | 0.970 |
| Abnormal CSF cells | 12 | 9 | 0.929 |
| Spinal cord involvement | 9 | 9 | 0.444 |
| Microhemorrhages | 26 | 21 | 0.224 |
| Cyclophosphamide | 25 | 15 | 0.937 |
| Azathioprine | 16 | 8 | 0.952 |
| Rituximab | 4 | 3 | 0.924 |
